# Storying health: the impact of illustrated narratives on children’s attitudes toward COVID-19 prevention in early education

**DOI:** 10.3389/fpsyg.2026.1803749

**Published:** 2026-03-23

**Authors:** Ruqaiya Hassan Al-Haddabi, Laila Al Salmi

**Affiliations:** 1 Government of Oman Ministry of Education, Muscat, Oman; 2 Department of Early Education, Sultan Qaboos University, Muscat, Oman

**Keywords:** covid-19 prevention, early childhood education, health literacy, illustrated stories, narrative pedagogy, Oman

## Abstract

This study examined the effectiveness of illustrated stories in promoting positive health attitudes related to COVID-19 prevention among fourth-grade students in Oman and explored students’ perceptions of using stories as tools for peer education. Guided by a mixed-methods explanatory sequential design, the study integrated quantitative and qualitative approaches to capture both behavioral outcomes and experiential insights. Forty-four fourth-grade students from Al-Shamael Basic Education School participated in a single-group pretest–posttest intervention involving four illustrated stories, The Dangerous Virus, The Smart Attack, Safe Return, and Protect Yourself, supported by a teacher guide emphasizing dialogic reading and active learning strategies. Data were collected using the COVID-19 Health Attitude Scale and semi-structured interviews with seven volunteer participants. Quantitative findings revealed a statistically significant increase in overall attitude scores from pretest (*M* = 2.27, SD = 0.41) to posttest (*M* = 2.49, SD = 0.38), *t*(43) = −3.77, *p* = 0.001, with notable improvement on the preventive behavior subscale. No significant gender differences were found, suggesting that story-based instruction engaged both male and female learners equitably. Thematic analysis of interview data identified three major themes: engagement and emotional connection, empowerment through peer teaching, and creativity in future story development. The convergence of findings indicates that illustrated stories provide a developmentally appropriate, culturally relevant, and emotionally resonant medium for health education in early primary grades. This study contributes novel evidence from the Omani context, demonstrating that illustrated narratives can serve as effective pedagogical tools for fostering health-promoting behaviors and advancing global health literacy goals in early childhood education.

## Introduction

A comprehensive understanding of COVID-19 and its transmission mechanisms is essential for preventing infection and mitigating the spread of disease. Within educational contexts, fostering such understanding among young learners represents both a pedagogical and public health priority. Schools serve as pivotal settings for cultivating foundational health literacy, and educators play a critical role in introducing developmentally appropriate information about communicable diseases. To be effective, these initiatives must employ instructional strategies that engage children’s cognitive, emotional, and social capacities. Among these strategies, illustrated stories emerge as a particularly promising medium for health education in early childhood.

Illustrated stories combine narrative structure, vivid imagery, and emotional resonance, thereby enhancing children’s comprehension and retention of abstract concepts (Rajab, 2017). Story-based instruction aligns closely with constructivist and socioemotional learning theories, which emphasize active meaning-making, imagination, and emotional engagement as pathways to knowledge construction (Bruner, 1990; Vygotsky, 1978). Research indicates that stories can facilitate both cognitive and affective learning by helping children relate to characters, visualize concepts, and internalize moral or behavioral lessons (Ahmed, 2009; Yamani et al., 2019). In the context of a global health crisis such as COVID-19, illustrated narratives can thus serve as effective vehicles for communicating complex health messages in accessible and meaningful ways.

Following the reopening of schools after pandemic-related closures, promoting hygienic habits among early elementary students became a pressing global concern. UNICEF, WHO, and UNESCO (2020) have underscored the need for comprehensive school-based health education that integrates disease prevention, handwashing, and sanitation practices into daily routines. However, the effectiveness of specific pedagogical strategies for achieving these aims in early childhood education remains underexplored. This gap is particularly evident in the Middle Eastern context, where few empirical studies have examined how illustrated stories might enhance children’s health-related attitudes and behaviors.

The spread of misinformation during the early stages of the COVID-19 pandemic further underscored the importance of accurate, age-appropriate health education (Qanshuba and Ra’ash, 2021). National health indicators, such as infection rates and system capacity, have emphasized the need for targeted educational interventions aimed at fostering informed and health-conscious behavior among young learners (Alhakawi et al., 2020). In this context, the present study examines the pedagogical potential of illustrated stories in promoting adherence to healthy habits related to COVID-19 prevention among fourth-grade students in the Sultanate of Oman. Specifically, it aims to assess (Al Salmi et al., 2022) the effectiveness of illustrated story-based instruction in developing positive health-related attitudes, to investigate possible gender-based differences in these attitudes, and to understand students’ perceptions of illustrated stories as tools for peer education and health promotion.

The study contributes theoretically by extending existing literature on early childhood health education and narrative-based pedagogy. Aligning with children’s cognitive and socioemotional development, it underscores the potential of illustrated stories as multimodal, evidence-based tools for integrating health communication and value formation in early learning environments. Practically, the study introduces a locally developed series of illustrated stories designed to promote health awareness and preventive behaviors related to COVID-19. These materials not only support children’s understanding of hygiene and disease prevention but also provide educators with a replicable framework for embedding public health messages within literacy instruction. To the researcher’s knowledge, this is the first empirical investigation in the Omani context to assess the efficacy of illustrated stories in enhancing young learners’ health-promoting attitudes and behaviors in response to a viral outbreak.

## Literature review

Global policy guidance during and after the COVID-19 pandemic positioned schools, especially early-years settings, as pivotal sites for health literacy and prevention. Joint WHO–UNICEF–UNESCO guidance called for risk-based approaches to school operations and the integration of age-appropriate hygiene and disease-prevention practices into daily routines (UNICEF, WHO, and UNESCO, 2020; WHO, 2020a, 2020b). UNICEF’s Key Messages and Actions for COVID-19 Prevention and Control in Schools specified concrete measures (e.g., hand hygiene, environmental cleaning, staying home when ill) and emphasized developmentally appropriate communication with children (UNICEF, WHO, and UNESCO, 2020). Complementing these operational directives, UNESCO urged systems to strengthen education’s role in public action and equitable recovery, underscoring the interdependence of education and health (UNICEF, WHO, and UNESCO, 2020). Within this policy frame, early-childhood educators require pedagogies that translate complex health information into accessible, engaging learning experiences.

### Theoretical foundations for narrative picture-book pedagogy

Two complementary theoretical strands explain the promise of illustrated stories (picture books, comics, and graphic-style narratives) for health education in young children. First, dual-coding theory posits that learning improves when information is encoded through coordinated verbal and nonverbal (visual) systems (Paivio, 1990). Picture books naturally leverage dual channels, scaffolding comprehension and memory for abstract concepts (e.g., germs, transmission). Second, narrative-transportation theory describes how immersion in story worlds can shape beliefs and attitudes by reducing counter-arguing and promoting identification with characters (Green and Brock, 2000). For young learners, relatable protagonists modeling health-protective routines (handwashing, masking during outbreaks, respiratory etiquette) can support internalization of norms through affective engagement.

These mechanisms align with socio-constructivist perspectives in early childhood: storybook discussions, prediction, and retelling constitute guided participation that helps children co-construct meanings with adults (Al-Salmi and Smith, 2015a, 2015b). A large body of evidence in early literacy shows that interactive/dialogic reading, teachers’ or caregivers’ open-ended questioning, feedback, and vocabulary scaffolding during shared reading, yields reliable gains in young children’s language outcomes (Al Salmi and Gelir, 2024; Wasik and Bond, 2001). Meta-analytic and experimental work further indicates that shared storybook reading enhances word learning, especially when visual load is optimized (e.g., fewer simultaneous illustrations) and books are re-read across days to consolidate learning (Flack et al., 2018; Horst et al., 2011; Flack and Horst, 2017). These design principles are directly translatable to health picture books that must introduce new vocabulary (e.g., “virus,” “vaccine,” “sanitize”) and causal reasoning about illness.

Beyond print, contemporary health messages for children often circulate through multimodal epitexts (book trailers, short videos, interactive snippets) that extend the storyworld and prime critical viewing/reading practices (Tabernero-Sala and Colón-Castillo, 2023). Analyses of 800 + picture-book “epitexts” show that combining image, voiceover, and short-form video can foreground key concepts and authorial intent, useful for introducing or reinforcing health content prior to or following shared reading sessions (Tabernero-Sala and Colón-Castillo, 2023).

Community-facing work also demonstrates that graphic-style stories read aloud in groups can strengthen health understanding for both children and caregivers. In a recent study published in Health Education and Behavior, reading short, co-designed, graphic narratives in community settings improved participants’ ability to interpret health information and discuss complex topics, with engagement supported by empathy for characters and narrative clarity (Kohli et al., 2023), which also affects program quality (Tekin et al., 2022).

### Evidence from COVID-19–focused narrative interventions

During COVID-19, several child-directed narrative initiatives translated public-health guidance into accessible stories. A widely disseminated example is “Leo and Giulia”, an Italian animated series created by public-health and media partners to explain SARS-CoV-2, mitigation measures, and later, vaccines to primary-school audiences (Vecchio et al., 2022; Odone et al., 2024). Although initial publications emphasize design, dissemination, and field-trial protocols rather than randomized impact evaluations, they offer a proof-of-concept for large-scale narrative health communication tailored to children.

Collectively, these COVID-era projects echo longstanding picture-book evidence: children benefit when health content is embedded in coherent stories with clear visuals, repeated exposure, and adult mediation. They also highlight the feasibility of co-design with public-health experts and media producers to ensure scientific accuracy and developmental appropriateness.

Synthesizing findings from early-literacy and health-communication research yields a set of practical design features for illustrated health stories aimed at young learners:

1. Visual parsimony and salience: Limit simultaneous illustrations and highlight the referent of new health vocabulary to support attention and mapping (Flack and Horst, 2017; Flack et al., 2018).
2. Repetition and spacing: Re-read stories across days and embed retrieval opportunities (retelling, enactment) to consolidate concepts (Horst et al., 2011).
3. Dialogic mediation: Use prompts (“Why did the character cover their cough?”) and contingent feedback to connect text to children’s experiences and school routines (Wasik and Bond, 2001).
4. Narrative identification: Feature age-proximal protagonists modeling target behaviors; leverage story arcs that make causal links between actions (e.g., handwashing) and outcomes (feeling safe at school), consistent with transportation mechanisms (Green and Brock, 2000).
5. Multimodal supports: Extend print stories with short videos or digital epitexts for preview/review and home–school continuity (Tabernero-Sala and Colón-Castillo, 2023).
6. Policy alignment and accuracy: Map story content to current school-health guidance (UNICEF, WHO, and UNESCO, 2020), updating editions as guidance evolves.

Despite promising evidence, the children’s health-story literature would benefit from (a) rigorous trials in school settings (cluster RCTs with behavioral outcomes), (b) mediator analyses testing whether transportation/identification explain attitude and behavior change in young children, and (c) longitudinal follow-up to examine maintenance of routines (e.g., hand hygiene) across grades. Additionally, equity-focused work should evaluate linguistic and cultural adaptations of stories in diverse contexts and assess family engagement when stories are sent home or read in community venues.

## Methodology and procedures

This study adopted a mixed-methods design to examine the effectiveness of illustrated stories in promoting positive health-related attitudes toward COVID-19 prevention among fourth-grade students, and to explore students’ perceptions of using these stories as tools for peer education. The design followed an explanatory sequential approach, beginning with a quantitative quasi-experimental phase followed by a qualitative phase to enrich interpretation. The single-group pretest–posttest design was selected due to ethical and contextual considerations. The illustrated stories were developed specifically for this study to promote COVID-19 preventive awareness. To ensure equitable access to potentially beneficial instructional materials, the intervention was delivered to the entire class rather than withholding it from a comparison group. Additionally, the study was conducted within one intact classroom, where random assignment was not feasible. The design was therefore appropriate for this exploratory investigation conducted in an authentic school setting. The qualitative phase was intentionally incorporated to enrich the interpretation of the quantitative findings and to provide deeper insight into students’ experiences with the intervention. This integration of methods enhanced the explanatory depth of the study, particularly given the limitations inherent in a single-group design. The study was conducted in October 2021 during the first semester of the 2021–2022 academic year, coinciding with the resumption of blended learning in Oman following COVID-19-related closures. The study included 44 fourth-grade students (23 boys and 21 girls) from Al-Shamael Basic Education School in the Al-Dakhiliyah Governorate, representing a purposive sample selected for accessibility and established collaboration with the researchers. While an *a priori* power analysis was not conducted due to the exploratory nature of the study and the use of an intact classroom sample, the within-subject pretest–posttest design increases statistical sensitivity by reducing between-subject variability. Observed effect sizes suggest adequate sensitivity for detecting meaningful changes in students’ attitudes. This school served as a suitable context given its emphasis on health and safety education during the pandemic. The broader population included all fourth-grade students in the governorate (N = 8,732), as documented in the Ministry of Education (2021). Seven students from the sample also volunteered to participate in follow-up interviews to share their perceptions of the intervention and its peer-learning potential.

The intervention consisted of four guided illustrated story sessions, each lasting approximately 30 min, delivered over 2 weeks. Each session included story reading, discussion, and reflective activities. The four illustrated stories, titled: The Dangerous Virus, The Smart Attack, Safe Return, and Protect Yourself, are each designed to communicate key health concepts aligned with the World Health Organization’s school health guidelines. The stories were written in Arabic and developed based on a review of evidence-based storytelling methods for young learners and were linguistically and visually adapted to reflect the developmental characteristics of early elementary students. To ensure intervention fidelity, all sessions followed a structured protocol including scripted story narration, guided discussion questions, and reflection prompts. A teacher guide accompanied the story series, providing lesson objectives, narrative prompts, and behavioral activities designed to reinforce understanding through discussion and demonstration. Each story session followed a similar format: teacher-led read-aloud, guided discussion using dialogic reading strategies, and a short practice activity such as demonstrating handwashing or mask-wearing. This structure integrates cognitive, social, and behavioral learning processes, consistent with constructivist and socioemotional theories of early childhood development. The first author observed all sessions and completed a fidelity checklist, confirming that the protocol was implemented consistently across sessions.

The primary quantitative instrument was the COVID-19 Health Attitude Scale, a 19-item measure developed by the researchers to assess students’ knowledge and preventive awareness related to COVID-19. Items were rated on a three-point Likert scale (1 = disagree, 2 = uncertain, 3 = agree). The instrument underwent expert validation by specialists in curriculum, psychology, and measurement to ensure content validity. Pilot testing with 30 students yielded item–subscale correlations ranging from 0.60 to 0.86 (*p* < 0.01), with Cronbach’s alpha coefficients of 0.405 for the knowledge subscale, 0.800 for preventive awareness, and 0.683 for the overall scale, acceptable for exploratory classroom-based research. The knowledge subscale consisted of nine items. Internal consistency analysis yielded a Cronbach’s alpha of 0.405. It is important to note that Cronbach’s alpha is sensitive to both the number of items and response scale variance. The use of a 3-point response format, appropriate for the developmental level of fourth-grade students, may have restricted response variability and attenuated inter-item correlations. The attitude subscale consisted of 12 items measuring positive attitudes toward COVID-19 prevention on a 5-point Likert scale.

Quantitative data were analyzed using SPSS. Descriptive statistics were computed for all variables, and pre–post differences were examined using paired-samples t-tests. Before conducting paired-sample t-tests, we examined the data for compliance with underlying assumptions. Normality of difference scores was assessed using the Shapiro–Wilk test, and visual inspection of histograms and Q–Q plots confirmed approximate normality. No extreme outliers were detected. These checks support the appropriateness of the parametric analyses. Where assumptions were not fully met, nonparametric alternatives (Wilcoxon signed-rank tests) were conducted and yielded consistent results.

Independent-samples t-tests were conducted to explore gender differences, with results interpreted alongside effect sizes to account for the modest sample size. Mean scores were interpreted as low (1.00–1.66), moderate (1.67–2.33), or high (2.34–3.00), in line with established scaling conventions.

The qualitative component employed semi-structured interviews designed to elicit students’ reflections on their engagement with the illustrated stories and their perceived usefulness as peer education tools. The interview protocol comprised four main questions and two follow-up prompts, reviewed by four academic experts to ensure clarity and developmental appropriateness. Individual interviews were conducted in a quiet classroom setting, lasted approximately 20–30 min, and were audio-recorded with permission. Transcripts were analyzed thematically using an inductive coding process to identify recurring ideas and patterns. Codes were clustered into higher-order themes, and analytic memos were maintained to enhance reliability and confirmability. The qualitative data were then integrated with the quantitative findings to provide a comprehensive understanding of how illustrated stories influenced students’ health-related attitudes and behaviors.

Ethical standards for research with children were strictly observed throughout the study. Permission to conduct the research was obtained from the school administration, and parents were informed of the study’s purpose and procedures. Participation was voluntary, and all students were assured that their responses would remain confidential and have no impact on their academic standing. Pseudonyms were used in all qualitative reporting to protect participant identity. Data were securely stored in password-protected files accessible only to the research team.

The analytic integration of quantitative and qualitative findings allowed for a more nuanced interpretation of results. Quantitative data identified the degree of attitudinal change following the intervention, while qualitative narratives provided insight into the specific story elements, characters, and activities that most effectively engaged students and reinforced learning. Together, these strands offer a holistic understanding of the potential of illustrated stories as a pedagogical strategy for promoting health literacy and preventive behavior among young learners in early educational settings.

## Results and discussion

The present study examined the effectiveness of illustrated stories in promoting fourth-grade students’ health-related attitudes toward COVID-19 prevention in Oman and explored their perceptions of using such stories as tools for peer education. An explanatory mixed-methods design was implemented, combining pre–post quantitative measures with qualitative interviews to provide a comprehensive understanding of how narrative-based instruction can enhance children’s health literacy and encourage preventive behaviors.

### Quantitative findings

Descriptive analyses indicated an overall improvement in students’ health-related attitudes following the illustrated story intervention. The mean score increased from *M* = 2.27 (SD = 0.41) in the pretest to *M* = 2.49 (SD = 0.38) in the posttest, demonstrating a shift from moderate to high agreement with statements reflecting positive health habits. Shapiro–Wilk tests indicated that difference scores for attitude subscales were approximately normally distributed (*p* > 0.05), supporting the use of paired t-tests. These results suggest a potential impact of exposure to the illustrated stories, which encouraged students to engage in preventive behaviors such as frequent handwashing, mask wearing, and maintaining personal hygiene. The increase is consistent with the developmental effectiveness of story-based learning reported in other early childhood contexts (Muthukrishnan, 2019; Yamani et al., 2019).

A paired-samples t-test confirmed that this increase was statistically significant, *t*(43) = −3.77, *p* = 0.001. The change represents a meaningful gain in students’ overall attitudes toward health-promoting practices. The illustrated stories used in the intervention incorporated familiar settings, simple dialogue, and colorful illustrations that appealed to children’s cognitive and emotional development, helping them translate abstract health messages into concrete understanding. As previous studies have found, such multimodal learning formats facilitate conceptual retention and behavioral imitation among young learners (Dakkak, 2012; Wang and Shao, 2025).

Subscale analysis showed that scores on the Preventive Behavior dimension improved significantly, whereas the Knowledge dimension remained relatively stable. This result indicates that while students already possessed foundational knowledge about COVID-19, most likely acquired from media, family, or school health campaigns, the illustrated stories may have contributed to behavioral intention and emotional engagement rather than factual recall. Similar findings have been documented in prior work emphasizing that children’s knowledge of pandemics tends to saturate early, whereas their consistent adoption of preventive behaviors depends on continued affective reinforcement and modeling (Al-Dmour et al., 2020; Gueron-Sela et al., 2023; Ahmed, 2016). Consequently, this study highlights the pedagogical potential of illustrated stories to transform existing knowledge into sustained, internalized behavior.

An independent-samples t-test was conducted to examine gender differences in pre- and post-test scores. Results showed no statistically significant differences between boys and girls at either time point, indicating that the illustrated stories were equally effective for both genders. This absence of gender disparity supports the argument that narrative-based instruction appeals universally to children through shared emotional and imaginative engagement (Hamdan, 2018; Syimah, 2017). In line with earlier work demonstrating gender-neutral benefits of story-based pedagogy (Zhang et al., 2025), this finding reinforces the inclusive nature of illustrated narratives as tools for early childhood health education.

The following (Table 1) shows the participating students’ attitudes toward COVID-19 before and after the intervention, displaying the mean and standard deviation of both knowledge and attitude scales.

**Table 1:** Students’ attitudes toward COVID-19 before and after the intervention (*N* = 44).

Statement	Sample	Agree (%)	Neutral (%)	Disagree (%)	Mean	SD	Level
Section 1: Knowledge of the COVID-19 Virus and Its Modes of Transmission							
I believe that **COVID-19** is an infectious virus.	Pre	63.6	20.5	15.9	2.48	0.76	High
	Post	88.6	4.5	6.8	2.82	0.54	High
I believe that infection with **COVID-19** harms our health.	Pre	63.6	11.4	25	2.39	0.87	High
	Post	93.2	0	6.8	2.86	0.51	High
I believe that everyone who gets **COVID-19 will** get severely harmed.	Pre	38.6	15.9	45.5	1.93	0.93	Medium
	Post	29.5	6.8	63.6	1.66	0.91	Low
I believe that infection with **COVID-19** occurs only through direct contact.	Pre	25	38.6	36.4	1.89	0.78	Medium
	Post	43.2	9.1	47.7	1.95	0.96	Medium
I believe that there is no need to dispose of tissue papers after using them.	Pre	29.5	22.7	47.7	1.82	0.87	Medium
	Post	25	2.3	72.7	1.52	0.88	Low
I think that gathering and playing with friends during the spread of **COVID-19** does not cause infection.	Pre	27.3	31.8	40.9	1.86	0.82	Medium
	Post	38.6	4.5	56.8	1.82	0.97	Medium
I believe that people infected with **COVID-19 s**how symptoms such as fever, shortness of breath, and coughing.	Pre	63.6	27.3	9.1	2.55	0.66	High
	Post	81.8	2.3	15.9	2.66	0.75	High
I believe that elderly people are more likely to be infected with **COVID-19.**	Pre	45.5	34.1	20.5	2.25	0.78	Medium
	Post	79.5	2.3	18.2	2.61	0.78	
I believe that we can see the **COVID-19 virus** with the naked eye.	Pre	22.7	29.5	47.7	1.75	0.81	Medium
	Post	40.9	4.5	54.5	1.86	0.98	Medium
Section 2: Health Awareness of Preventive Measures Against COVID-19							
I make sure to wear a **mask** when leaving home.	Pre	77.3	6.8	15.9	2.61	0.75	High
	Post	84.1	6.8	9.1	2.75	0.61	High
I avoid shaking hands with family and friends when meeting them.	Pre	61.4	18.2	20.5	2.41	0.82	High
	Post	79.5	6.8	13.6	2.66	0.71	High
I make sure to sanitize my hands when touching surfaces.	Pre	65.9	13.6	20.5	2.45	0.82	High
	Post	88.6	4.5	6.8	2.82	0.54	High
I make sure to wash my hands with **soap and water** to avoid infection with **COVID-19**.	Pre	75	18.2	6.8	2.68	0.60	High
	Post	93.2	4.5	2.3	2.91	0.36	High
I avoid touching my eyes directly with my hands.	Pre	38.6	25	36.4	2.02	0.88	Medium
	Post	68.2	6.8	25	2.43	0.87	High
I make sure to keep a distance of **at least 1 m** between myself and others.	Pre	68.2	15.9	15.9	2.52	0.76	High
	Post	86.4	6.8	6.8	2.80	0.55	High
I maintain my **personal hygiene** regularly.	Pre	56.8	27.3	15.9	2.41	0.76	High
	Post	88.6	6.8	4.5	2.84	0.48	High
I prevent my classmate from using my **personal belongings** during the spread of **COVID-19.**	Pre	52.3	18.2	29.5	2.23	0.89	Medium
	Post	84.1	4.5	11.4	2.73	0.66	High
I make sure to wear **gloves** when entering commercial establishments.	Pre	59.1	22.7	18.2	2.41	0.79	High
	Post	86.4	2.3	11.4	2.75	0.65	High
I warn my classmates about the dangers of **gathering at school so** that we do not get infected with **COVID-19.**	Pre	68.2	13.6	18.2	2.50	0.79	High
	Post	88.6	0	11.4	2.77	0.64	High
Overall	Pre	52.80%	21.70	25.60	2.27	0.31	Medium
	Post	72.00	4.50	23.40	2.49	0.26	High

The overall results of the scale demonstrate a positive change in participants’ attitudes and awareness toward COVID-19 following the intervention. The overall mean score increased from *M* = 2.27 (SD = 0.31) in the pre-test to *M* = 2.49 (SD = 0.26) in the post-test. In addition, the percentage of agreement increased from 52.8% in the pre-test to 72.0% in the post-test.

These findings indicate a shift from a moderate level of awareness in the pre-measurement to a high level in the post-measurement, suggesting that the intervention was effective in improving participants’ knowledge and health awareness regarding COVID-19 and its preventive measures.

### Qualitative findings

The semi-structured interviews provided rich and nuanced insight into how students perceived and interacted with the illustrated stories. Thematic analysis identified three major, interconnected themes: engagement and emotional connection, empowerment through peer teaching, and creativity with future-oriented learning.

All interviewed students described the stories as enjoyable, memorable, and easy to understand. Many indicated that the visual depictions of the virus and preventive behaviors helped them overcome fear and confusion. One student commented, “We now know what the virus looks like,” while another remarked, “The story taught us how to protect ourselves and what steps to follow.” These statements illustrate how stories can convert complex or abstract information into concrete, emotionally resonant messages, aligning with constructivist and socioemotional learning theories emphasizing the role of meaning-making and emotional safety in young children’s comprehension (Bruner, 1990; Vygotsky, 1978).

Students also expressed strong motivation to share the illustrated stories with others, revealing an emerging sense of agency as health communicators. They proposed retelling the stories during class breaks, morning assemblies, and even on school YouTube channels. One child suggested dramatizing the stories in a theatrical performance, highlighting how narrative learning extends beyond individual comprehension into social participation. This aligns with findings by Kohli et al. (2023) and Tympa and Karavida (2021), who showed that collaborative storytelling fosters empathy, cooperation, and community engagement among children.

A third theme involved creativity and future-oriented learning. Students generated ideas for writing their own stories, suggesting topics such as the daily experiences of doctors, caring for family members during illness, and the consequences of ignoring health advice. These proposals demonstrate metacognitive awareness and reflect children’s capacity to generalize story-based lessons into novel contexts. Such creative engagement confirms that illustrated stories can stimulate higher-order thinking and imagination, consistent with research highlighting the role of narrative in fostering critical reflection and adaptive reasoning (Tabernero-Sala and Colón-Castillo, 2023; Bruner, 1990).

## Discussion

While the findings suggest that illustrated stories may be a promising tool for promoting positive health-related attitudes, the results should be interpreted cautiously due to the study design. The findings are interpreted within the context of the study sample, which consisted of 44 students from a single school. While the results provide insight into the potential value of illustrated stories in this setting, they may not be generalizable to broader populations without replication across diverse educational contexts.

The convergence of quantitative and qualitative findings strengthens confidence in the interpretation that illustrated stories may represent a developmentally appropriate and pedagogically valuable approach to promoting positive health attitudes among young learners. The significant improvement in posttest scores supports the interpretation that illustrated narratives may help bridge cognitive and affective learning domains within this context. The simultaneous use of imagery and text engages both visual and verbal processing systems, consistent with dual coding theory (Paivio, 1990), which enhances memory and conceptual understanding. Furthermore, students’ absorption into the narrative and identification with the characters reflect narrative transportation processes (Green and Brock, 2000), offering a plausible explanation for how affective engagement may contribute to positive attitudinal shifts that are theoretically associated with behavior. These findings make a novel contribution to the existing literature by providing empirical evidence from an underrepresented regional context. Few studies in the Middle East or North Africa have examined narrative-based approaches to health education in early primary settings. By embedding international health guidelines from the UNICEF, WHO, and UNESCO (2020) within locally contextualized illustrated materials, this research illustrates how global health literacy objectives can be meaningfully localized. It thereby addresses a critical gap in early childhood education research, namely, the lack of culturally responsive, evidence-based models for integrating health promotion into foundational learning.

The results also extend theoretical discussions of narrative pedagogy by confirming its dual function: as a mechanism for knowledge retention and as a catalyst for social participation. Illustrated stories, when implemented through guided reading and reflective discussion, not only enhance comprehension but also promote social–emotional learning and civic responsibility. Similar integrative benefits have been observed in story-based programs addressing environmental awareness (Rajab, 2017; Hamdan, 2018), nutrition (Widiana and Harwanto, 2025), and community safety (Rashwan, 2017).

This study provides preliminary empirical support for the potential value of illustrated stories as a holistic pedagogical tool that unites literacy, health, and social development in early childhood education. Students demonstrated improved attitudes following exposure to the illustrated stories toward health-promoting behaviors, showing that narrative, imagery, and guided discussion may facilitate the translation of knowledge into action and strengthen intentions and readiness to engage in health-promoting behaviors. Beyond health education, these findings affirm that storytelling remains one of the most powerful modes of instruction for young learners because it integrates emotion, cognition, and social meaning.

From a practical standpoint, illustrated story interventions can be embedded across subjects such as language arts, social studies, and life skills to strengthen both literacy and health literacy outcomes. Teachers can enhance impact by employing dialogic reading strategies and by facilitating reflective discussion to connect story content with students’ daily experiences. Policymakers and curriculum designers are encouraged to collaborate with educators, artists, and public health professionals to develop culturally relevant illustrated stories addressing a range of contemporary health issues.

Future research should extend this work through longitudinal studies to evaluate behavioral persistence and through cross-cultural comparisons to explore contextual adaptability. Incorporating digital and interactive story formats, such as e-books and animation, may further enhance engagement and allow for broader dissemination. As schools continue to navigate the post-pandemic landscape, illustrated narratives offer an evidence-based, developmentally grounded, and emotionally resonant strategy to strengthen children’s health awareness, empathy, and social responsibility.

### Conclusion and implications

This research provides encouraging evidence suggesting that illustrated stories may represent a promising pedagogical approach for enhancing children’s health-related attitudes and behaviors in early educational contexts. The observed improvement in students’ overall attitudes toward COVID-19 prevention suggests that story-based learning has the potential to bridge the gap between cognitive understanding and behavioral application. By integrating narrative, imagery, and guided interaction, illustrated stories activate multiple learning modalities: visual, linguistic, and emotional; that support the internalization of preventive behaviors. These findings align with dual coding theory (Paivio, 1990), which posits that information encoded through both verbal and visual channels enhances memory retention, and with narrative transportation theory (Green and Brock, 2000), which explains how emotional engagement with stories increases persuasion and greater openness toward preventive behaviors.

The findings indicate similar patterns of attitudinal change across genders, suggesting that illustrated narratives appeal universally to children’s sense of curiosity and imagination. Qualitative insights revealed that students not only understood and remembered the health messages but also felt empowered to share them with peers and family members, illustrating the potential of narrative-based instruction to foster social participation and peer learning. These findings are particularly relevant in the post-pandemic educational landscape, where fostering children’s agency, empathy, and resilience is as critical as transmitting factual information (Kohli et al., 2023; Tympa and Karavida, 2021).

The study makes a distinctive contribution by providing region-specific evidence from Oman, a context with limited empirical research on narrative-based health education in early primary settings. By embedding international health recommendations from the UNICEF, WHO, and UNESCO (2020) within culturally contextualized illustrated stories, the study demonstrates how global health literacy initiatives can be localized through child-centered pedagogy. The research thus addresses a critical gap by empirically validating narrative learning as a means to promote public health behaviors among young learners in Arabic-speaking educational systems.

The implications for practice are substantial. Illustrated stories can be systematically integrated into early childhood curricula within subjects such as language arts, social studies, and life skills to strengthen both literacy and health competencies. Teacher preparation programs should incorporate training on dialogic reading, story facilitation, and cross-curricular health education. Schools and education ministries may also collaborate with illustrators, authors, and public health experts to develop localized story materials addressing current health challenges in accessible and engaging ways. However, given the absence of a comparison group, these findings should be interpreted as context-specific and exploratory, providing a foundation for future controlled investigations.

Future research should employ longitudinal and comparative designs to examine the sustainability of behavioral changes and the transfer of narrative-based health learning across contexts. Expanding this line of inquiry to include digital and interactive story formats could reveal additional pathways for engagement, particularly in technology-rich classrooms. By positioning illustrated stories at the intersection of literacy, health, and socioemotional development, this study contributes to the growing international discourse on narrative as a catalyst for holistic learning and wellbeing in early childhood education.

### Limitations of the study

The study relied on a quasi-experimental pre-post scale design with one experimental group. Although this design is widely used in educational research to organize educational applications and evaluate their impact, it lacks a control group with which to compare results. The absence of a control group restricts the possibility of attributing changes in attitudes solely to the independent variable (the storyboards) without other external influences such as moods or unexpected events in the educational environment (such as repetition effects in pretest scale performance).

The relatively low internal consistency of the knowledge subscale may reflect the limited response variability inherent in a 3-point Likert scale and the developmental characteristics of the sample. Future research may benefit from expanding the response range or increasing item numbers to enhance reliability.

It is important to note that the study assessed self-reported attitudes rather than observed behaviors. Therefore, conclusions should be limited to short-term attitudinal change, and future research should examine whether these shifts translate into sustained behavioral outcomes.

The relatively small sample size and the inclusion of students from a single school limit the generalizability of the findings. Future research should replicate the study across multiple schools and regions to strengthen external validity.

Although an *a priori* power analysis was not conducted due to the exploratory nature of the study and the use of an intact classroom sample, the observed effect sizes suggest adequate statistical sensitivity for detecting within-group changes. Nevertheless, future studies with larger samples are recommended to confirm these findings.

## Data Availability

The raw data supporting the conclusions of this article will be made available by the authors, without undue reservation.
